# A novel macrolide–Del-1 axis to regenerate bone in old age

**DOI:** 10.1016/j.isci.2024.108798

**Published:** 2024-01-04

**Authors:** Kridtapat Sirisereephap, Hikaru Tamura, Jong-Hyung Lim, Meircurius Dwi Condro Surboyo, Toshihito Isono, Takumi Hiyoshi, Andrea L. Rosenkranz, Yurie Sato-Yamada, Hisanori Domon, Akari Ikeda, Tomoyasu Hirose, Toshiaki Sunazuka, Nagako Yoshiba, Hiroyuki Okada, Yutaka Terao, Takeyasu Maeda, Koichi Tabeta, Triantafyllos Chavakis, George Hajishengallis, Tomoki Maekawa

**Affiliations:** 1 Division of Periodontology, Graduate School of Medical and Dental Sciences, Niigata University, Niigata 951-8514, Japan; 2 Center for Advanced Oral Science, Graduate School of Medical and Dental Sciences, Niigata University, Niigata 951-8514, Japan; 3 Faculty of Dentistry, Chulalongkorn University, Bangkok 10330, Thailand; 4 Department of Basic and Translational Sciences, Laboratory of Innate Immunity and Inflammation, School of Dental Medicine, University of Pennsylvania, Philadelphia, PA 19104, USA; 5 Faculty of Dentistry, Universitas Airlangga, Surabaya 60132, Indonesia; 6 Division of Microbiology and Infectious Diseases, Graduate School of Medical and Dental Sciences, Niigata University, Niigata 951-8514, Japan; 7 Ōmura Satoshi Memorial Institute, Kitasato University, 5-9-1 Shirokane, Minato-ku, Tokyo 108-8641, Japan; 8 Graduate School of Infection Control Sciences, Kitasato University, 5-9-1 Shirokane, Minato-ku, Tokyo 108-8641, Japan; 9 Division of Cariology, Operative Dentistry and Endodontics, Department of Oral Health Science, Graduate School of Medical and Dental Sciences, Niigata University, Niigata 951-8514, Japan; 10 Laboratory of Clinical Biotechnology, Center for Disease Biology and Integrative Medicine, Graduate School of Medicine, The University of Tokyo, Tokyo 113-8655, Japan; 11 Institute for Clinical Chemistry and Laboratory Medicine, University Hospital and Faculty of Medicine, Technische Universität Dresden, Dresden, Germany; 12 Centre for Cardiovascular Science, Queen’s Medical Research Institute, University of Edinburgh, Edinburgh, UK

**Keywords:** Immunology, Age, Small molecule

## Abstract

Aging is associated with increased susceptibility to chronic inflammatory bone loss disorders, such as periodontitis, in large part due to the impaired regenerative potential of aging tissues. DEL-1 exerts osteogenic activity and promotes bone regeneration. However, DEL-1 expression declines with age. Here we show that systemically administered macrolide antibiotics and a non-antibiotic erythromycin derivative, EM-523, restore DEL-1 expression in 18-month-old (“aged”) mice while promoting regeneration of bone lost due to naturally occurring age-related periodontitis. These compounds failed to induce bone regeneration in age-matched DEL-1-deficient mice. Consequently, these drugs promoted DEL-1-dependent functions, including alkaline phosphatase activity and osteogenic gene expression in the periodontal tissue while inhibiting osteoclastogenesis, leading to net bone growth. Macrolide-treated aged mice exhibited increased skeletal bone mass, suggesting that this treatment may be pertinent to systemic bone loss disorders. In conclusion, we identified a macrolide–DEL-1 axis that can regenerate bone lost due to aging-related disease.

## Highlights

• Macrolides and EM-523 increase DEL-1 in the gingiva and periodontium of aged mice
• Macrolides and EM-523 promote bone regeneration in old age
• Macrolides and EM-523 suppress osteoclastogenesis *in vivo* and *in vitro*
• Macrolides and EM-523 induces osteogenesis *in vivo* and *in vitro*

## Graphical abstract

**Figure:**
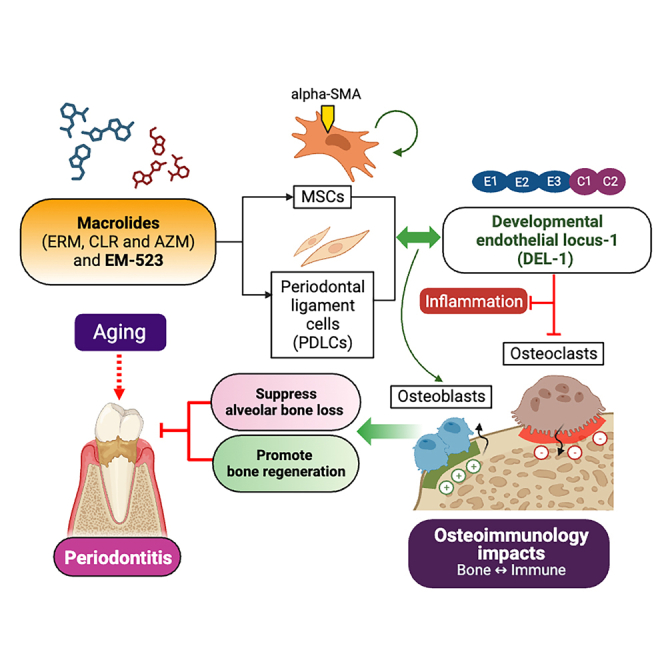

## Introduction

Periodontitis is a dysbiotic inflammatory disease affecting the integrity of the periodontium, tooth-supporting tissues that include the gingiva, periodontal ligament (PDL), and alveolar bone.^1^ Untreated periodontal disease may lead to tooth loss and systemic complications, creating a serious public health and economic burden.^2^^,^^3^^,^^4^^,^^5^^,^^6^ There is an unmet need for immune modulation therapies with some promising approaches currently in clinical development.^7^^,^^8^ This health issue is particularly evident in elderly individuals, who have increased susceptibility to and prevalence of not only periodontal disease but other bone loss disorders, such as osteoporosis.^9^^,^^10^^,^^11^^,^^12^^,^^13^

The association of periodontitis with aging does not simply reflect the cumulative effect of prolonged exposure to the dysbiotic microbiome of the periodontal pockets. Aging may increase susceptibility to periodontitis by altering the immune inflammatory status and/or the regenerative potential of the periodontal tissues.^10^^,^^14^^,^^15^^,^^16^^,^^17^ Old age is associated with increased periodontal disease severity not only in humans but also in mice, which are thus a useful model to study the impact of aging on this oral bone loss disorder.^11^^,^^15^^,^^18^^,^^19^ In both humans and mice, aging has been associated with altered periodontal stem and progenitor cell function in the perivascular niche of the PDL; these altered functions can impair the reparative capacity of periodontal tissue, including regeneration of alveolar bone lost due to periodontitis.^14^^,^^20^^,^^21^^,^^22^^,^^23^^,^^24^

Development endothelial locus-1 (DEL-1) is a homeostatic protein secreted by tissue-resident cells in the gingiva and the PDL, endothelial and mesenchymal stromal/stem cells (MSCs) and some macrophage subsets, and contributes to inflammation resolution and tissue repair.^25^ Specifically, during the resolution phase of experimental periodontitis in mice, DEL-1 promotes efferocytosis and the emergence of the macrophage pro-resolving phenotype as well as stimulating alveolar bone regeneration.^26^^,^^27^ The pro-regenerative function of DEL-1 is largely independent of its efferocytic/pro-resolving function and involves activation of a β3 integrin–FAK–ERK1/2–RUNX2 pathway in osteoprogenitor cells.^27^ Alveolar bone regeneration fails in DEL-1-deficient mice or in mice that express a DEL-1 point mutant that cannot bind β3 integrins.^27^ The expression of DEL-1 is severely diminished in old age, both in mice and humans.^28^^,^^29^

Given that DEL-1 levels decline severely in old age and mice ≥18 months of age are DEL-1 deficient,^30^ it is important to develop potential therapeutic approaches to stimulate DEL-1 expression, thereby restoring the levels of this important homeostatic protein in the elderly. We have recently shown that the macrolide antibiotic erythromycin (ERM)—but not other antibiotics, such as penicillin and josamycin—stimulates the production of DEL-1 in vascular endothelial cells. Specifically, erythromycin interacts with the growth hormone secretagogue receptor (GHSR) and activates JAK2 and p38 MAPK signaling, leading to C/EBPβ-dependent DEL-1 expression.^31^ Moreover, systemic erythromycin treatment in mice increased DEL-1 expression in the PDL, which connects the tooth to the surrounding alveolar bone.^31^ The PDL of humans and animals, including mice, contains a mostly perivascular MSC niche involved in periodontal tissue regeneration and includes progenitor cells that can differentiate into osteoblasts.^14^^,^^22^^,^^32^^,^^33^^,^^34^^,^^35^

We show that erythromycin and other macrolides restore DEL-1 expression in old mice and promote regeneration of bone lost due to naturally occurring, aging-related periodontitis. The same treatment increased the bone mass in the femurs of old mice. Importantly, EM-523, a non-antibiotic derivative of erythromycin, that retains the ability to activate a homolog of the GHSR, motilin receptor,^36^^,^^37^ reproduced the DEL-1-dependent effect of erythromycin on bone regeneration. Mechanistically, macrolide and EM-523 treatments induced the formation of new bone by upregulating alkaline phosphatase (ALP) activity and the expression of osteogenic genes in periodontal tissue while reducing the number of osteoclasts, thereby favorably influencing the osteogenesis/osteoclastogenesis balance. Periodontal bone lost due to periodontitis has limited capacity for regeneration even after standard treatment (scaling and root planing) and surgical periodontal therapy, especially in elderly patients.^38^^,^^39^^,^^40^ Therefore, the non-antibiotic compound EM-523 may represent a safe, effective, and affordable new approach to regenerate bone lost due to periodontitis in humans and perhaps for increasing the mineral content of the skeletal bone in the elderly.

## Results

### Local treatment with DEL-1-Fc induces bone regeneration in 12-month-old mice

**Figure 1:**
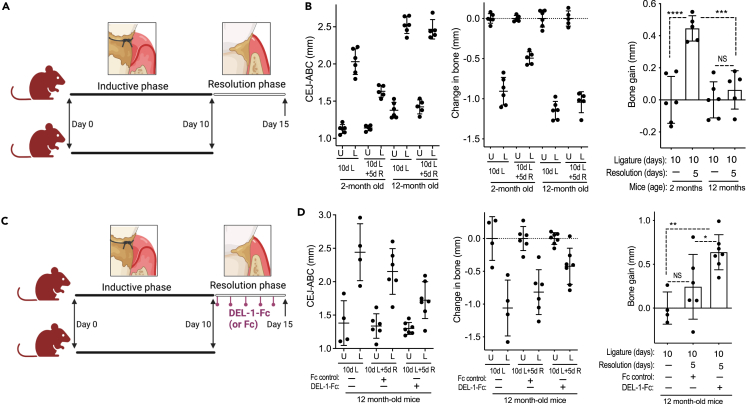
Local treatment with DEL-1-Fc promotes bone regeneration in 12-month-old mice (A) Experimental design. (B, left) Measurement of bone heights (distance from cement-enamel junction [CEJ] to alveolar bone crest [ABC]; CEJ-ABC) in groups of 2-month-old and 12-month-old mice after ten days of ligature placement (10dL) or after 10 days of ligature placement followed by five days without ligatures to enable resolution from periodontitis (10dL + 5dR). (B, middle) Data from the left panel were transformed to show bone loss at ligated (L) sites vs. unligated (U) contralateral sites. (B, right) Data from the middle panel were transformed to show bone growth relative to corresponding 10dL group (baseline). (C) Experimental design. (D) 12-month-old mice were subjected to LIP for ten days with or without ligature removal for five days to enable resolution, with or without local microinjection with DEL-1-Fc (1 μg) or equal molar amount of Fc control. Treatments were performed daily on days 10–14. (D, left) Measurement of bone heights (CEJ-ABC distance). (D, middle) Data from the left panel were transformed to show bone loss in ligated (L) sites vs. unligated (U) contralateral sites. (D, right) Data from the middle panel were transformed to indicate bone growth relative to the 10dL group (baseline). Data are means ± S.D. (B, n = 5–6 mice/group; D, n = 4–7 mice/group). ∗p < 0.05, ∗∗p < 0.01; one-way ANOVA and Dunnett’s test.

We have previously monitored DEL-1 expression in the periodontal tissue of mice of different ages (ranging from 5 weeks to 24 months of age) and observed a gradual reduction in DEL-1 expression with advancing age, with the oldest mice becoming essentially DEL-1 deficient.^30^ In the present study, we first investigated whether aging affects bone regeneration in mice. Aging and its impact on tissue regeneration capacity is a gradual process, and an aging-related phenotype may manifest itself before a mouse is of formal “geriatric age,” ≥18 months.^11^ We first evaluated 12-month-old mice in a proof-of-concept experiment, using our previously established model, in which bone loss was induced by silk ligature placement (ligature-induced periodontitis, LIP) at the left maxillary second molar for ten days followed by ligature removal for five days to allowDEL-1-dependent inflammation resolution and bone regeneration.^27^ Whereas young (2-month-old) mice readily regenerated bone upon ligature removal, the 12-month-old mice did not exhibit significant bone regeneration (Figures 1A and 1B). However, when the 12-month-old mice were locally microinjected in the gingiva with recombinant DEL-1-Fc, bone regeneration was observed (Figures 1C and 1D). These data suggest that DEL-1 treatment can rescue the aging-related defect in bone regeneration.

### Macrolides induce bone regeneration in a DEL-1-dependent manner in 18-month-old mice with naturally occurring periodontitis

In a model of aging-related periodontal bone loss, it is not practical to perform daily microinjections of DEL-1-Fc as performed in the experiment in Figure 1. Moreover, given the high cost of recombinant protein production, an affordable approach with a convenient delivery method would be desirable. Since we have previously shown that erythromycin upregulates DEL-1 expression in the periodontal tissue of young mice,^31^ we investigated whether erythromycin and other macrolides DEL-1 can induce bone regeneration in 18-month-old mice thereby promoting the regeneration of bone lost due to aging-related periodontitis. Macrolides were administered by intraperitoneal injection since direct palatal injection of antibiotics can exert antimicrobial effects on the ligature-associated microbiota, thereby confounding the results.^41^ The microbial load extracted from the ligatures was comparable between control- and macrolide-treated groups suggesting that peritoneal injection of the macrolides did not exert an inhibitory effect on the microbiota (Figure S1).

**Figure 2:**
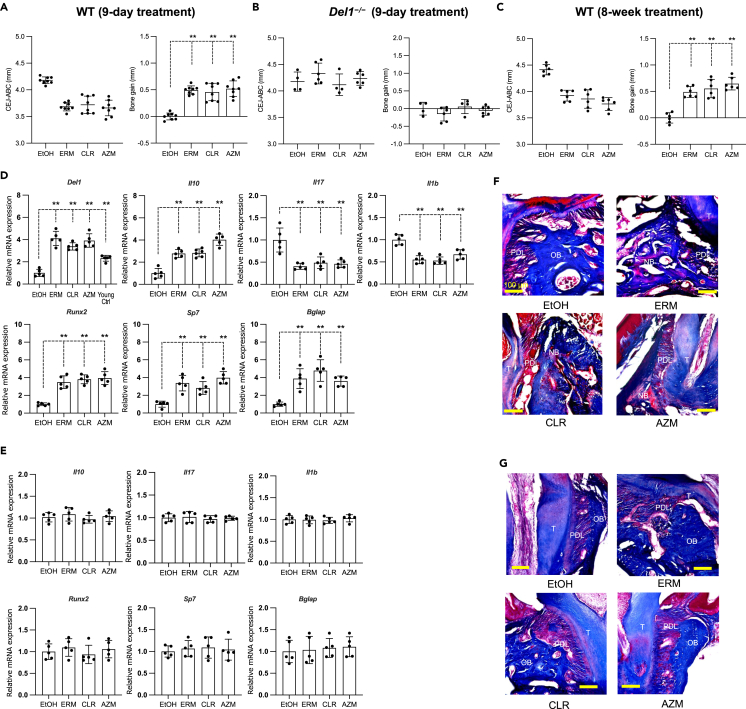
Macrolides induce bone regeneration in a DEL-1-dependent manner in 18-month-old mice with naturally occurring periodontitis (A and B) Aged WT and aged *Del1*^*−/−*^ mice were administered macrolide antibiotics or vehicle control (ethanol) intraperitoneally daily for nine days. (C) Aged WT were administered macrolide antibiotics intraperitoneally twice a week for eight weeks. (A–C) The distance from the CEJ-ABC was measured (left panels). The CEJ-ABC data were transformed to show the bone changes relative to the control, which was set as the baseline (right panels). Positive values (in mm) indicate bone growth relative to the baseline (control). (D and E) Analysis of gene expression from gingiva samples collected from (D) aged WT and (E) aged *Del1*^*−/−*^ mice after nine days of drug treatment. The mRNA levels of the indicated genes were quantified by qPCR. Data were normalized to *Gapdh* mRNA and plotted relative to the aged control group, set as 1. (F and G) Coronal maxillary sections from (F) aged WT and (G) aged *Del1*^−/−^ mice were stained with modified Masson’s trichrome stain, which stains the mature (old) bone *blue* and new bone formation *red*. T: tooth; PDL: periodontal ligament; OB: old bone as *blue;* NB*:* new bone as *red*. Scale bars, 100 μm. Data are means ± SD (A, n = 8 mice/group; B, n = 4–6 mice/group; C, n = 6 mice/group; D-E, n = 5 mice/group). ∗∗p < 0.01; one-way ANOVA and Dunnett’s test.

Mice develop naturally occurring periodontal bone loss, which becomes severe at an age ≥15 months.^19^ Groups of mice, aged 19 to 20-month-old and randomly distributed into control and experimental groups were treated with erythromycin, clarithromycin, or azithromycin (ERM, CLR, and AZM, respectively) by daily intraperitoneal injection for nine days. Macrolide-treated aged mice exhibited significantly reduced bone heights (CEJ-ABC distances) relative to the control group (Figure 2A, left panel). When the data were transformed to show a change in bone levels relative to the control serving as baseline, the macrolide-treated aged mice displayed positive values, indicating bone regeneration (Figure 2A, right panel). However, this regenerative effect was not observed in macrolide-treated aged *Del1*^−/−^ mice (Figure 2B). Indeed, the CEJ-ABC distances of the macrolide- and control-treated aged *Del1*^*−/−*^ mice were comparable (Figure 2B, left panel), indicating no bone regeneration due to macrolide treatment (Figure 2B, right panel). To study the long-term effects of macrolide treatment, aged mice were systemically treated with a macrolide twice a week for eight weeks (Figure 2C). Similar to the shorter treatment, aged mice treated with macrolides for eight weeks showed significantly reduced bone heights relative to the control-treated aged mice, (Figure 2C, left panel) indicating bone regeneration (Figure 2C, right panel). These results suggest that macrolides rapidly promote bone regeneration in aged wild-type (WT) mice in a DEL-1-dependent manner. To further support this notion, we examined the effects of macrolide treatment on DEL-1 expression and stimulation of osteogenic activity in the periodontal tissue of aged WT mice.

Quantitative real-time PCR (qPCR) of RNA extracted from the palatal gingiva of aged WT mice with naturally occurring periodontitis showed that all macrolides tested significantly upregulated the gene expression of *Del1* and anti-inflammatory gene, *Il10*. Macrolides suppressed the expression of pro-inflammatory genes *Il17* and *Il1b* (Figure 2D, upper panel). Moreover, all macrolides tested significantly upregulated key osteoblast differentiation-related genes, *Runx2*, *Sp7*, and *Bglap*, involved in the early, middle, and late phases of osteoblast differentiation, respectively (Figure 2D, lower panel). In contrast, macrolide treatment did not downregulate inflammatory genes (Figure 2E, upper panel) or upregulate osteoblast-related genes in the gingiva of aged *Del1*^−/−^ mice (Figure 2E, lower panel). The data shown in Figures 2A and 2B suggest that aged WT, but not *Del1*^*−/−*^, mice grow new bone upon macrolide treatment. To further support this notion, we stained maxillary sections using modified Masson’s trichrome stain. Histological observations revealed new bone formation in the macrolide-treated groups in aged WT mice (Figure 2F). Consistent with a requirement for DEL-1 expression, no new bone formation was observed in the tissue sections from macrolide-treated aged *Del1*^*−/−*^ mice (Figure 2G).

Together, these findings suggest that treatment of aged mice with macrolides promotes inflammation resolution, upregulation of osteogenic genes, and bone regeneration in a macrolide-induced *Del1*-dependent manner.

### Macrolides induce DEL-1 protein expression in the PDL of aged periodontium and promote the binding of C/EBPβ to the *DEL1* promoter

**Figure 3:**
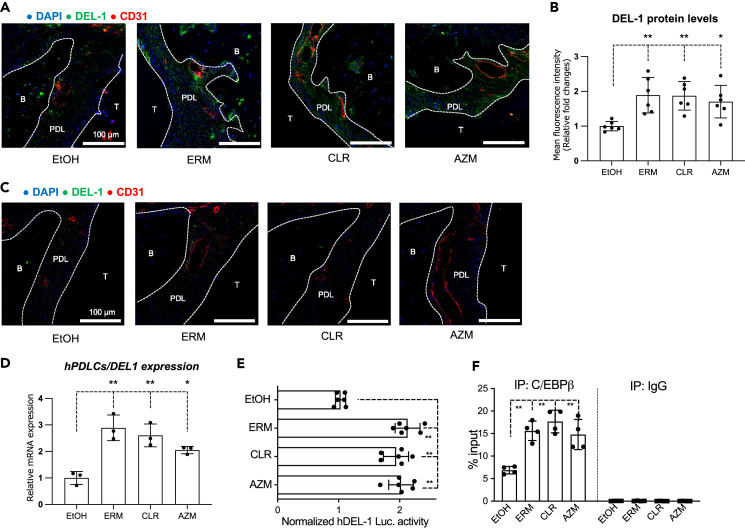
Macrolides induce DEL-1 protein expression in PDL spaces of the aged periodontium and promote the binding of C/EBPβ to the *DEL1* promoter Aged WT and aged *Del1*^*−/−*^ mice were administered macrolide antibiotics or vehicle control (ethanol) intraperitoneally daily for nine days. (A) Representative IF images of maxillary sections from aged WT mice obtained with fluorescence microscopy. Frozen maxillae sections were stained for DEL-1 (green), CD31 (red), and the nuclei were counterstained using DAPI (blue). T: Tooth; PDL: periodontal ligament; B: alveolar bone. Scale bars, 100 μm. (B) Mean fluorescence intensity (MFI) values were measured from randomly selected IF images from aged WT mice using the ImageJ software. All MFI values were normalized and plotted relative to the MFI of the control group, set as 1. (C) Representative IF images of maxillary sections from aged *Del1*^*−/−*^ mice obtained with fluorescence microscopy. Frozen maxillae sections were stained for DEL-1 (green), CD31 (red), and the nuclei were counterstained using DAPI (blue). T: tooth; PDL: periodontal ligament; B: alveolar bone. Scale bars, 100 μm. (D) The mRNA levels of *DEL1* from hPDLCs were quantified by qPCR on day seven of the drug administration. Data were normalized to *GAPDH* mRNA and plotted relative to the aged control group, set as 1. (E) hPDLCs were transiently transfected with hEDIL3-promoter-Luc reporter plasmid, pre-treated for 30 min with ethanol control, or indicated macrolides (10 μg/mL), and analyzed for luciferase activity. A renilla luciferase construct was co-transfected as an internal control for normalization. Data are calculated as fold change relative to ethanol control treatment, which was set as 1. (F) ChIP analysis of C/EBPβ binding capacity at the *DEL1* promoter in hPDLCs treated for 4 h with ethanol control or indicated macrolides (10 μg/mL). Non-immunoprecipitated cell extracts were used as input samples. Data are plotted as a percentage of input. Data are means ± SD (B, n = 6 mice/group; D, n = 3 sets of cultures/group; E, n = 6 sets of cultures/group; F, n = 4 sets of cultures/group). ∗p < 0.05, ∗∗p < 0.01; one-way ANOVA and Dunnett’s test.

We next determined whether macrolides promote DEL-1 protein expression. We performed immunofluorescence (IF) to detect DEL-1 in maxillary tissue sections of aged WT mice focusing on the PDL, where MSCs proliferate and undergo osteogenic differentiation, leading to new alveolar bone formation.^35^^,^^42^ The results showed diminished DEL-1 protein expression in the PDL of control-treated aged mice; however, DEL-1 protein levels in the PDL were significantly elevated in macrolide-treated aged mice compared to those in the control group (Figures 3A and 3B). Staining for DEL-1 in the PDL of aged *Del1*^*−/−*^ mice confirmed the absence of DEL-1regardless of control or macrolide treatment (Figure 3C).

To obtain insights into the mechanism of macrolide-induced DEL-1 expression in the PDL, We examined the effect of macrolide-treatment on hPDLCs, which contain MSCs. All three macrolides tested, ERM, CLR, AZM, upregulated *DEL1* mRNA expression above the baseline levels of the control group (Figure 3D). Additionally, hPDLCs transfected with an hDEL1-promoter-Luc-plasmid and treated with macrolides or control showed a significant upregulation of *DEL1* promoter activity by all macrolides compared to the control treatment (Figure 3E). We previously showed that the transcription factor C/EBPβ regulates *DEL1* transcription and that ERM enhances its binding to the *DEL1* promoter in endothelial cells.^43^ To examine whether a similar mechanism operates in hPDLCs, we performed a chromatin immunoprecipitation assay (ChIP-qPCR). In all macrolide-treated groups, we detected C/EBPβ enrichment at the *DEL1* promoter, in contrast to control samples (Figure 3F). Together, these data suggest that the increased DEL-1 gene and protein expression in the PDL of macrolide-treated aged mice could be attributed, at least in part, to macrolide stimulation of *DEL1* transcription by promoting C/EBPβ binding to the *DEL1* promoter.

### Macrolides promote osteogenic activity and reduce osteoclastogenesis in the PDL of aged WT but not *Del1*^−/−^ mice

**Figure 4:**
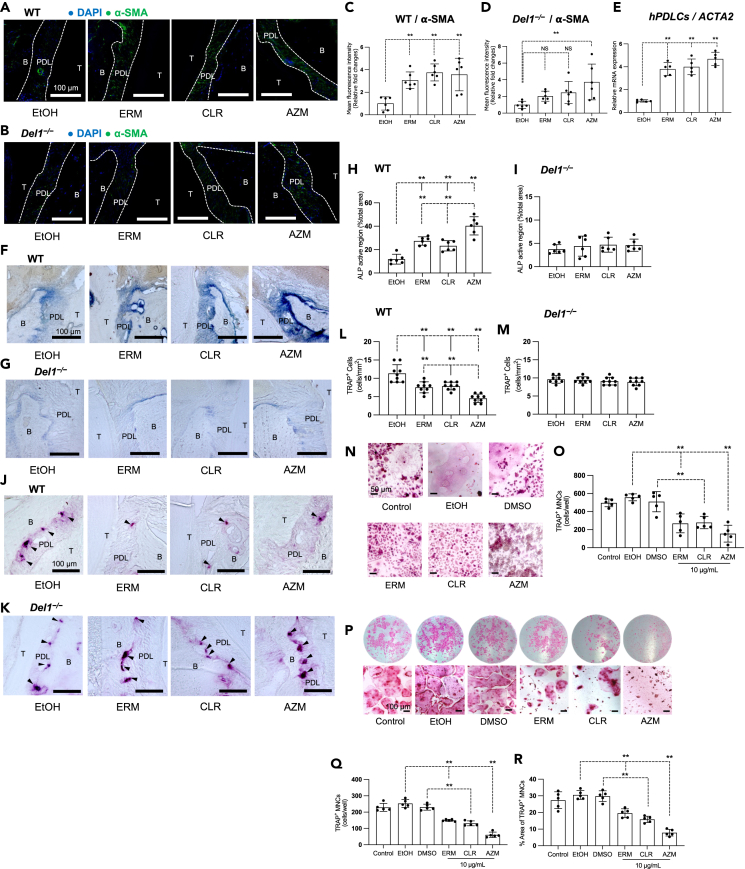
Macrolides promote osteogenic activity and reduce osteoclastogenesis in the PDL space of aged WT but not *Del1*^−/−^ mice Aged WT and aged *Del1*^*−/−*^ mice were administered macrolide antibiotics or vehicle control (ethanol) intraperitoneally daily for nine days. (A and B) Representative IF images of maxillary sections obtained by fluorescence microscopy. Frozen maxillae sections were stained for alpha-smooth muscle actin (α-SMA, green), and the nuclei were counterstained using DAPI (blue). T: tooth; PDL: periodontal ligament; B: alveolar bone. Scale bars, 100 μm. (C and D) Mean fluorescence intensity (MFI) values were measured in IF images from aged WT and *Del1*^−/−^ mice using the ImageJ software. All MFI values were normalized and plotted relative to the MFI of the control group in aged mice, set as 1. (E) Cultures of hPDLCs were treated with ethanol control or indicated macrolides (10 μg/mL). The mRNA levels of *ACTA2* from hPDLCs were quantified by qPCR on day seven after drug administration. Data were normalized to *GAPDH* mRNA and plotted relative to the aged control group, set as 1. (F and G) Optical microscopy images of maxillary sections show PDL areas stained for ALP activity. Maxillary sections are from (F) aged WT and (G) aged *Del1*^***−/−***^ mice. T: tooth; PDL: periodontal ligament; B: alveolar bone. Scale bars, 100 μm. (H and I) The ALP-positive region of each area was quantified and represented as a percentage of the total area. (J and K) Optical microscopy images of maxillary sections show PDL areas stained for TRAP. Maxillary sections were obtained from (J) aged WT and (K) aged *Del1*^**−/−**^ mice. Arrowheads indicate TRAP^+^ cells in the PDL space. T: tooth; PDL: periodontal ligament; B: alveolar bone. Scale bars, 100 μm. (L and M) The number of TRAP^+^ cells in the PDL space were counted and compared between indicated groups. (N and O) RAW264.7 cells were treated with RANKL for one week to induce osteoclast differentiation. (N) Representative images of TRAP^+^ MNCs. Scale bars, 50 μm. (O) The number of TRAP^+^ MNCs was counted. (P–R) Bone marrow cells harvested from C57BL/6 mice were subjected to RANKL-induced osteoclastogenesis assay in the presence of macrolides (10 μg/mL) or control solvent for one week. (P) Representative images of TRAP^+^ MNCs on day seven of the assay. Scale bars, 100 μm. (Q) The number of TRAP+ MNCs was counted after staining with TRAP. (R) Calculation of the percentage area of TRAP^+^ cells were performed using ImageJ software. Data are means ± SD (C–D, n = 6 mice/group; E, n = 5 sets of cultures/group; H–I, 6 mice/group; L–M, n = 8–9 mice/group; O, Q, R, n = 5 sets of cultures/group).∗∗p < 0.01; (C–E, one-way ANOVA and Dunnett’s test; H-I, one-way ANOVA and Tukey’s test; O, Q, R, one-way ANOVA and Bonferroni’s test).

The ability of the PDL MSCs to proliferate declines with age, resulting in impaired bone regeneration.^21^^,^^24^ α-Smooth muscle actin (α-SMA) is commonly found in vascular smooth muscle cells and myofibroblasts, which arise from mesenchymal cells during tissue injury and repair.^44^ α-SMA is a marker for identifying osteoprogenitor cells in MSC populations.^35^ Increased expression of α-SMA leads to cell contraction and serves as a crucial factor in diminishing the clonal capacity of human MSCs (hMSCs) and influencing their differentiation toward osteoblasts.^45^ To further study the effect of macrolide treatment on promoting osteogenic differentiation, we used α-SMA as a selective marker for osteoprogenitor cells from MSCs. IF images of maxillary tissue sections detecting α-SMA revealed increased α-SMA protein levels in all macrolide-treated groups of aged WT mice relative to the control (Figures 4A and 4C). In aged *Del1*^−/−^ mice, only AZM, of the three macrolides tested, appeared to increase the protein expression of α-SMA in the PDL (Figures 4B and 4D). In hPDLCs ERM, CLR, and AZM upregulated the mRNA expression of the α-SMA-encoding gene (*ACTA2*) relative to ethanol control (Figure 4E). The association of macrolide treatment with increased mRNA and protein expression of α-SMA further supports the notion that macrolides promote osteogenic activity in the PDL.

Macrolide-induced promotion of bone regeneration in aged mice suggests that the macrolides favorably regulate the osteogenesis-osteoclastogenesis balance. To test this, we performed ALP staining (Figures 4F–4I) and tartrate-resistant acid phosphatase (TRAP) staining (Figures 4J–4M) on maxillary tissue sections to detect osteogenesis and osteoclastogenesis, respectively, on the alveolar bone surfaces in the PDL of aged WT and *Del1*^**−/−**^ mice treated with macrolides or control. The analyses showed that macrolide treatment resulted in increased ALP activity (Figures 4F and 4H) and decreased numbers of osteoclasts (Figures 4J and 4L) in aged WT mice but not in aged *Del1*^**−/−**^ mice, in which macrolide treatment had no effect (Figures 4G, 4I, 4K, and 4M, for ALP and osteoclasts, respectively). AZM appeared to strongly promote ALP activity and suppress osteoclastogenesis compared to ERM and CLR (Figures 4H and 4L). Together the bone measurements (Figure 2A) modified Masson’s trichrome staining (Figure 2F), and the increased ALP activity in macrolide-treated aged mice indicate that macrolide treatment promotes osteoblast activity which leads to bone regeneration in aged WT mice.

The ability of macrolides to reduce the number of osteoclasts *in vivo* in a DEL-1-dependent manner could, in part, be attributed to their anti-inflammatory effects. Another potential mechanism may involve direct inhibitory effects on osteoclastogenesis, given that DEL-1 was shown to directly regulate osteoclast differentiation.^46^ To study the effect of macrolide treatment on osteoclast differentiation, we performed an osteoclastogenesis assay using the RAW264.7 mouse cell line. RAW264.7 cells were treated with macrolides at a concentration well below the cytotoxic concentration (Figure S2). Macrolide-treated RAW264.7 cells yielded significantly fewer TRAP^+^ MNCs than control-treated RAW264.7 cells (Figures 4N and 4O). Similar inhibitory effects on osteoclastogenesis by all three macrolides were observed when we used bone marrow-derived monocyte/macrophage precursor cells^47^ in a RANKL-induced osteoclast differentiation assay (Figures 4P–4R). Among the three macrolides tested, AZM displayed the most potent inhibitory effect on osteoclastogenesis (Figures 4P–4R). These data suggest that macrolides, in particular AZM, inhibit RANKL-induced osteoclastogenesis *in vitro* and reduce the number of osteoclasts in the aged periodontium *in vivo*.

In summary, *in vivo* studies show that macrolides promote osteogenic and restrain osteoclastogenic activity in the PDL of aged mice in a DEL-1-dependent manner. Consistently, these macrolide effects are replicated in relevant *in vitro* assays.

### ERM increases the bone mass and affects osteolineage cell parameters of mouse femurs

**Figure 5:**
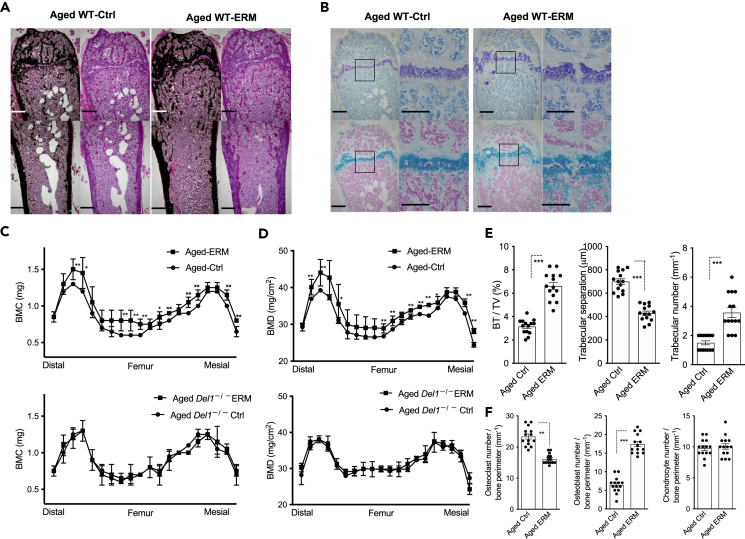
ERM increases the bone mass and affects the osteolineage cell parameters of mouse femurs (A–F) Aged WT mice were administered with ethanol control or ERM intraperitoneally twice a week for eight weeks. (A and B) Representative images of Von Kossa (A), Toluidine blue (B, upper panel), Alcian blue (B, lower panel)-stained femur. Scale bars, 500 μm. (C and D) Bone mineral content (BMC) and BMD of femurs measured by DXA. (E) Microstructural parameters of the distal femurs. BV/TV, bone volume per total volume. (F) Osteolineage cells parameters measured from Von Kossa staining images. Data are means ± SD (C and D n = 4 mice/group; E,F n = 14 mice/group). ∗p < 0.05, ∗∗p < 0.01; Student’s t test.

To determine whether the pro-osteogenic effects of macrolide treatment also impact the skeletal bone, we next analyzed the femurs of control- and ERM-treated aged mice. ERM-treated aged mice showed a significant increase in bone mass compared with age-matched controls (Figures 5A–5D). Bone morphometric analysis revealed that ERM treatment elevated bone volume fraction (BV/TV), trabecular number, and reduced trabecular separation (Figure 5E). ERM treatment also affected osteolineage cell parameters within mouse femurs. The femurs from ERM-treated aged mice exhibited an increased number of osteoblasts and decreased number of osteoclasts, while chondrocytes were not significantly affected, as compared to the control group (Figure 5F). Together, these data suggest that ERM treatment promotes bone regeneration in old mice not only in the periodontium but also in long tubular bones throughout the body.

### EM-523, a non-antibiotic erythromycin derivative, promotes bone regeneration in aged mice and *in vitro* studies

The overuse of antibiotics and increasing incidence of antimicrobial resistance has prompted researchers to dissociate the antimicrobial effects of antibiotics from their anti-inflammatory and immunomodulating functions, which could be exploited for treating inflammatory diseases. EM-523 is an erythromycin derivative that is devoid of antimicrobial properties^4^^,^^35^ but can activate the motilin receptor, a homolog of GHSR,^36^^,^^37^ which we have shown mediates ERM-induced DEL-1 upregulation.^31^ We investigated whether EM-523 could reproduce the previously described bone regenerative effects of ERM.

**Figure 6:**
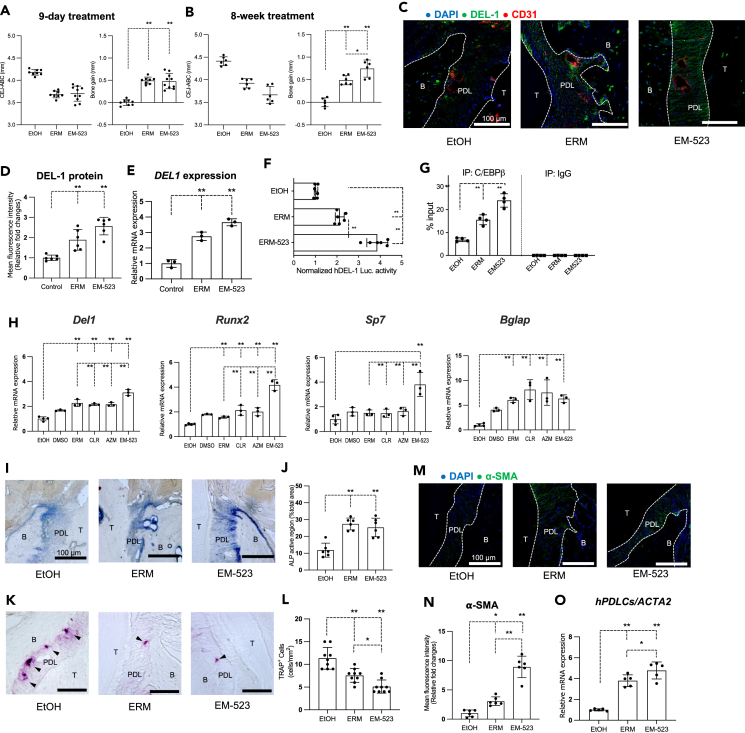
EM-523, a non-antibiotic erythromycin derivative, promotes bone regeneration in aged mice (A and B) Aged WT mice were treated with ethanol control, ERM, or EM-523 intraperitoneally (A) daily for nine days or (B) twice a week for eight weeks. The distance from the CEJ-ABC was measured (left panels). The CEJ-ABC data were transformed to show the bone changes relative to the control, which was set as the baseline (right panels). Positive values (in mm) indicate bone growth relative to the baseline (control). (C) Representative IF images of maxillary sections obtained by fluorescence microscopy. Frozen maxillae sections were stained for DEL-1 (green), CD31 (red), and the nuclei were counterstained using DAPI (blue). T: tooth; PDL: periodontal ligament; B: alveolar bone. Scale bars, 100 μm. (D) Mean fluorescence intensity (MFI) values of DEL-1 were measured from randomly selected IF images from aged WT using the ImageJ software. All MFI values were normalized and presented relative to the MFI of the control group in aged WT mice, set as 1. (E) The mRNA levels of *DEL1* from human periodontal ligament cells (hPDLCs) were quantified by qPCR on day seven after drug treatment. Data were normalized to *GAPDH* mRNA and plotted relative to the aged control group, set as 1. (F) hPDLCs were transiently transfected with hDEL1-promoter-Luc reporter plasmid, pre-treated for 30 min with ethanol control, ERM (10 μg/mL), or EM-523 (1 μg/mL), and analyzed for luciferase activity. A renilla luciferase construct was co-transfected as an internal control for normalization. Data were calculated as fold change relative to ethanol control treatment, which was set as 1. (G) ChIP analysis of C/EBPβ binding capacity at the DEL1 promoter in hPDLCs treated for 4 h with ethanol control, ERM, or EM-523 (10 and 1 μg/mL, respectively). Non-immunoprecipitated cell extracts were used as input samples. Data are plotted as a percentage of input. (H) Gene expression analysis using qPCR of primary mouse PDLCs on day nine of the osteogenic differentiation assay for the expression of the indicated genes. Cells were treated with vehicle control, macrolides, or EM-523 (ERM, CLR and AZM as 10 μg/mL; EM-523 as 1 μg/mL). Data were normalized to *Gapdh* mRNA and plotted relative to ethanol-treated control, set as 1. (I–N) Aged WT mice were treated with ethanol control, ERM, or EM-523 intraperitoneally for nine days. (I) Optical microscopy images of WT maxillary sections show PDL areas stained for ALP activity. T: Tooth; PDL: periodontal ligament; B: alveolar bone. Scale bars, 100 μm. (J) The ALP-positive region of each area was quantified and represented as a percentage of the total area. (K) Optical microscopy images of WT maxillary sections show PDL areas stained for TRAP. Arrowheads indicate TRAP+ cells in PDL space. T: Tooth; PDL: periodontal ligament; B: alveolar bone. Scale bars, 100 μm. (L) The number of TRAP^+^ cells within the PDL space were counted and compared between indicated groups. (M) Representative IF images of maxillary sections obtained by fluorescence microscopy. Frozen maxillary sections were stained for alpha-smooth muscle actin (α-SMA, green), and the nuclei were counterstained using DAPI (blue). T: tooth; PDL: periodontal ligament; B: alveolar bone. Scale bars, 100 μm. (N) Mean fluorescence intensity (MFI) values were measured from IF images using the ImageJ software. All MFI values were normalized and plotted relative to the MFI of the control group in aged mice, set as 1. (O) Cultures of hPDLCs were treated with ethanol control, ERM, or EM-523 (10 and 1 μg/mL). The mRNA levels of *ACTA2* from hPDLCs were quantified by qPCR on day seven after drug treatment. Data were normalized to *GAPDH* mRNA and plotted relative to the aged control group, set as 1. Data are means ± S.D. (A, n = 8–10 mice/group; B, n = 6 mice/group; D, n = 6 mice/group; E, n = 3 sets of cultures/group; F, n = 6 sets of cultures/group; G, n = 4 sets of cultures/group; H, n = 3–4 sets of cultures/group; J, n = 6 mice/group; L, n = 9 mice/group; N, n = 6 mice/group; O, n = 5 sets of cultures/group). ∗p < 0.05, ∗∗p < 0.01; (one-way ANOVA and Tukey’s test; only H, one-way ANOVA and Bonferroni’s test).

Aged mice (19 to 20-months-old) treated with EM-523 for nine days exhibited significantly reduced bone heights (Figure 6A, left panel), indicating bone regeneration compared to the control-treated aged mice (Figure 6A, right panel). The effect of EM-523 was comparable to that of ERM (Figure 6A). However, when EM-523 and ERM were tested in an eight-week treatment regimen, EM-523-treated aged mice exhibited significantly greater bone regeneration relative to ERM-treated aged mice (Figure 6B).

IF detecting DEL-1 in maxillary sections from experimental and control-treated aged mice showed that EM-523 and ERM comparably promoted DEL-1 protein expression in the PDL relative to control-treated aged mice (Figures 6C and 6D). Furthermore, in hPDLCs cultures, EM-523 upregulated *DEL1* gene expression relative to treatment with the control (Figure 6E). Moreover, we transfected hPDLCs with a hDEL1-promoter-Luc-plasmid and treated them with ERM, EM-523, or control. Measurements of luciferase activity showed a significant upregulation of *DEL1* promoter activity in EM-523-treated mice compared to both ERM and control treatments (Figure 6F). To examine whether EM-523 promotes C/EBPβ binding to the *DEL1* promoter in hPDLCs, ChIP-qPCR was conducted and showed that EM-523 showed higher C/EBPβ enrichment at the *DEL1* promoter than both the ERM-treated and the control-treated groups (Figure 6G).

qPCR of RNA extracted from primary mouse PDLCs, which underwent osteogenic differentiation for nine days, revealed that EM-523 induced stronger upregulation of *Del1* gene expression as compared to not only the control but to all macrolides tested (Figure 6H). Similarly, compared to the macrolides tested, EM-523 exhibited the most potent effect on promoting *Runx2* and *Sp7* expression. Additionally, EM-523 significantly upregulated *Bglap* compared to the vehicle-control group (Figure 6H).

Given that EM-523 promotes DEL-1 expression and bone regeneration, we next determined if EM-523 could promote a pro-osteogenic environment in the PDL, in side-by-side experiments with ERM. Maxillary tissue sections of aged mice treated with control, ERM, or EM-523, were stained for ALP (Figures 6I and 6J) and TRAP (Figures 6K and 6L) to detect, osteogenic and osteoclastogenic activity, respectively, on the alveolar bone surfaces in the PDL. Relative to the control treatment, but similar to ERM, the EM-523 treatment resulted in increased ALP activity (Figure 6J). The EM-523 treatment reduced the number of osteoclasts in the PDL of the aged periodontium as compared to both ERM and control groups (Figure 6L). IF images of maxillary tissue sections detecting α-SMA revealed increased α-SMA protein levels in EM-523-treated aged mice relative to both ERM and control (Figures 6M and 6N). Moreover, in hPDLCs cultures, EM-523 upregulated *ACTA2* gene expression relative to both ERM and control treatments (Figure 6O).

**Figure 7:**
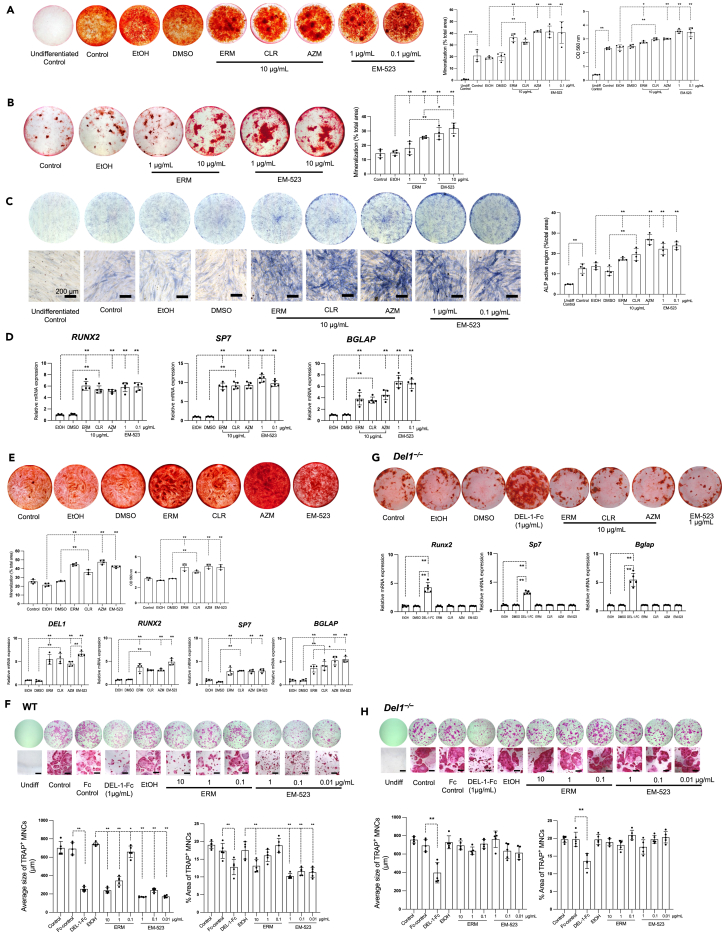
EM-523 promotes bone regeneration and suppresses osteoclastogenesis *in vitro* (A) MC3T3-E1 cells were cultured in osteogenic differentiation medium treated with control solvent or indicated macrolides. (A, left panel) Representative images of mineralization nodules from osteoblasts, stained with Alizarin Red S (ARS) staining after 26 days of the osteogenic differentiation assay. (A, middle panel) The mineralization area in each culture was quantified and represented as a percentage of the total area. (A, right panel) Quantification of the ARS staining using 10% cetylpyridinium chloride (CPC) on day 26 of the osteogenic differentiation assay. The absorbance was measured at 560 nm. The control group was incubated with an osteogenic differentiation medium only. (B) MC3T3-E1 cells were cultured in osteogenic differentiation medium treated with ethanol, ERM, or EM-523. (B, left panel) Representative images of mineralization nodules from osteoblasts, stained with ARS after 20 days of the osteogenic differentiation assay. (B, right panel) The mineralization area in each culture was quantified and presented as a percentage of the total area. The control group was incubated with an osteogenic differentiation medium only. (C) hPDLCs were cultured in osteogenic differentiation medium with control solvent or indicated macrolides. (C, left panel) Representative images of hPDLCs cultures, stained with alkaline phosphatase stain after 26 days of the osteogenic differentiation assay. Scale bars, 200 μm. (C, right panel) The ALP-positive region of each culture was quantified and represented as a percentage of the total area. (D) Gene expression analysis of hPDLCs on day nine of the osteogenic differentiation assay using qPCR. *RUNX2*, *SP7*, and *BGLAP*, as representative markers for early, middle, and late osteogenic markers, respectively. Data were normalized to *GAPDH* mRNA and plotted relative to ethanol-treated control, set as 1. (E) hMSCs were cultured in osteogenic differentiation medium with ethanol or indicated macrolides (10 μg/mL). (E, upper panel) Representative images of mineralization nodules from osteoblasts, stained with Alizarin Red S after 26 days of the osteogenic differentiation assay. (E, middle left panel) The mineralization area in each culture was quantified and presented as a percentage of the total area. (E, middle right panel) Quantification of the Alizarin Red S staining using 10% CPC on day 26 of the osteogenic differentiation assay. (E, lower panel) Gene expression analysis using qPCR of human MSCs on day nine of the osteogenic differentiation assay for the expression of the indicated genes. Data were normalized to *GAPDH* mRNA and plotted relative to ethanol control, set as 1. (F) Bone marrow cells were collected from WT mice for the osteoclastogenesis assay. Cells were treated with DEL-1-Fc (1 μg/mL), equal molar amount of Fc control, control solvent, or indicated macrolides for one week. (F, upper panel) Representative images of the culture well from each group after TRAP staining are shown. Histological images of TRAP^+^ MNCs. Scale bars, 200 μm. (F, lower left panel) The average size of TRAP^+^ MNCs was measured using the ImageJ software. (F, lower right panel) The percentage of TRAP^+^ area per total area was measured using the ImageJ software. (G) Primary osteoblastic progenitor cells were collected from *Del1*^*−/−*^ mice. Primary osteoblastic progenitor cells were cultured in osteogenic differentiation media administered with DEL-1-Fc, equal molar amount of Fc control, control solvent, or indicated macrolides for 26 days. (G, upper panel) Representative images of mineralization nodules from osteoblasts, stained with Alizarin Red S after 26 days of the osteogenic differentiation assay. (G, lower panel) Gene expression analysis was completed on day nine of the osteogenic differentiation assay using qPCR. *Runx2*, *Sp7*, and *Bglap*, as representative markers for early, middle, and late osteogenic markers, respectively. Data were normalized to *GAPDH* mRNA and plotted relative to ethanol control, set as 1. (H) Bone marrow cells were collected from *Del1*^*−/−*^ mice for the osteoclastogenesis assay. Cells were treated with DEL-1-Fc, equal molar amount of Fc control, control solvent, or indicated macrolides for one week. (H, upper panel) Representative images of the culture well from each group after TRAP staining were shown. Histological images of TRAP^+^ MNCs. Scale bars, 200 μm. (H, lower left panel) The average size of TRAP^+^ MNCs was measured using the ImageJ software. (H, lower right panel) The percentage of TRAP^+^ area per total area was measured using the ImageJ software. Data are means ± S.D. (A–C, n = 4 sets of cultures/group; D, n = 5 sets of cultures/group; E middle and lower panel, n = 4 sets of cultures/group; F–H, n = 5 sets of cultures/group). ∗p < 0.05, ∗∗p < 0.01; one-way ANOVA and Bonferroni’s test.

Consistent with its *in vivo* effects on osteoblasts and osteoclasts, EM-523 induced osteogenic differentiation in MC3T3-E1 cells (Figures 7A and 7B), hPDLCs (Figures 7C and 7D) and human iPSC-derived MSCs (Figure 7E), as well as inhibited RANKL-induced osteoclast differentiation from primary bone marrow-derived osteoclast progenitor cells (Figure 7F). EM-523-treated sampled showed increased calcified nodule formation in MC3T3-E1 cultures compared to ERM (Figures 7A and 7B). Similarly, EM-523 treatment increased ALP activity (Figure 7C, right panel) and upregulated the expression of osteoblast-related genes (*RUNX2*, *SP7*, and *BGLAP*) in hPDLC-differentiated osteoblasts (Figure 7D) at lower concentrations than those used for ERM and other macrolides. Moreover, when human iPSC-derived MSCs cultured in osteogenic medium for 26 days were treated with EM-523 or other macrolides, all compounds increased calcified nodule formation. In addition, the expression of *DEL1* and osteogenic genes *RUNX2*, *SP7*, and *BGLAP* were upregulated under the same conditions (Figure 7E). In contrast, treatment with macrolides and EM-523 did not upregulate osteoblast-related genes in osteoblastic progenitor cells derived from *Del1*^−/−^ mice (Figure 7G). In RANKL-induced osteoclastogenesis, EM-523 reduced the size of osteoclasts and percentage of the TRAP^+^ area, similarly to ERM and DEL-1-Fc. When compared to ERM, EM-523 exerted its inhibitory effect even at very low concentrations (≤0.1 μg/mL) (Figure 7F, lower panel). However, the inhibitory effect of EM-523 on osteoclastogenesis was not observed in the osteoclastogenesis assay using primary osteoclast progenitors from *Del1*^−/−^ mice (Figure 7H), indicating that the action of EM-523 is DEL-1-dependent. Under DEL-1-deficient conditions, only DEL-1-Fc treatment could reduce the size of osteoclasts and the percentage of the TRAP^+^ area (Figure 7H, lower panel).

These data suggest that EM-523 is a stronger inducer of DEL-1 mRNA and protein expression than ERM. By increasing the expression of DEL-1, EM-523 stimulates osteoblastic activity while suppressing osteoclastogenesis, thereby promoting bone growth in aged mice.

## Discussion

Different approaches have been proposed to regenerate bone lost due to periodontal disease. These include surgical implantation of scaffolds, allogeneic or autologous bone material, the use of stem cells, and treatment with bone morphogenetic proteins and growth factors. However, there is currently limited evidence on the predictability and efficacy of these emerging regenerative therapies to warrant routine clinical application.^48^^,^^49^^,^^50^^,^^51^^,^^52^ An affordable and effective regenerative strategy may involve approaches that promote tissue responses to favorably modulate endogenous stem cell niches and tissue microenvironments, thereby enhancing tissue repair and regeneration, which are impaired in old age.^53^ In the present study, we have made a significant conceptual and translational advance by establishing a macrolide–DEL-1 axis that stimulates bone regeneration in old age. Specifically, we have shown that macrolides, and, importantly, a non-antibiotic derivative, can restore the expression of the homeostatic and pro-osteogenic protein DEL-1,^25^^,^^27^^,^^46^ the expression of which declines with aging in both mice and humans.^28^^,^^29^ The aging-related decline in DEL-1 expression coincides with increased susceptibility to and prevalence of periodontitis and other inflammatory bone loss disorders.^9^^,^^10^^,^^11^^,^^12^ Therefore, restoring DEL-1 protein levels with an affordable and safe approach holds a great promise for treating elderly patients.

The association of old age with increased susceptibility to chronic inflammatory conditions, including periodontal disease, may be due to alterations in the immune and inflammatory status of aging tissues combined with a reduction in the regenerative ability of PDL cells.^10^^,^^14^^,^^15^^,^^54^ The PDL contains a heterogeneous mesenchymal stromal and stem cell population responsible for the regeneration of periodontal tissue, including the extracellular matrix and adjacent mineralized tissues, such as the alveolar bone. In this context, fibroblasts, which produce the extracellular matrix, and multipotent stem cells, which can differentiate into osteoblasts that form alveolar bone, are morphologically indistinguishable and may share cell surface markers.^55^^,^^56^^,^^57^^,^^58^^,^^59^^,^^60^^,^^61^ Multipotent human PDL cells can undergo osteogenic, adipogenic, or chondrogenic differentiation depending on the culture conditions and can regenerate periodontal tissue upon transplantation to immunodeficient mice.^32^^,^^34^^,^^62^^,^^63^ The ability of the PDL MSCs to proliferate and undergo osteogenic differentiation declines with age, resulting in impaired tissue repair.^20^^,^^21^^,^^24^ This aging-related dysfunction may not be entirely due to intrinsic MSC changes but may be attributed to alterations in the niche microenvironment.^20^^,^^21^^,^^24^^,^^64^^,^^65^^,^^66^^,^^67^^,^^68^^,^^69^^,^^70^ Such aging-related dysfunctional alterations may also involve the declining expression of DEL-1 by tissue-resident cells, including vascular endothelial cells.^28^^,^^71^

The perivascular MSC niche in the PDL contributes critically to periodontal connective tissue and bone regeneration.^22^^,^^32^^,^^33^^,^^34^^,^^72^^,^^73^^,^^74^ DEL-1 expressed in the PDL niche may not only contribute to the resolution of inflammation^26^ but can also induce osteoblastogenesis and the formation of new alveolar bone during inflammation resolution.^27^ Therefore, the aging-related deficiency of DEL-1 may contribute to the failure of the aged niche to respond to the need for tissue repair and bone regeneration. The failure of the aged tissue to properly repair and regenerate itself can be reversed, as we show in this paper, by restoring the levels of DEL-1 in the PDL using macrolides and a non-antibiotic derivative, EM-523. In perivascular areas, the PDL contains a progenitor population, expressing the MSC markers STRO-1 and CD146, that can generate multiple mesenchymal lineages, including fibroblasts, cementoblasts, and osteoblasts.^22^^,^^32^^,^^35^^,^^62^ Here, we showed that macrolides and EM-523 upregulate the expression of DEL-1 not only in the PDL of aged mice, but also in cultures of hMSCs, which, in the presence of macrolides, undergo enhanced osteogenic differentiation. DEL-1, endogenously produced in hMSCs, might regulate their proliferation and/or differentiation toward osteoblasts. Since the macrolide treatment was administered systemically, it is likely that the macrolides modulated MSCs at sites different from the PDL. Consistent with this notion, the systemic treatment of aged mice with ERM, twice a week for eight consecutive weeks, resulted in increased bone mass in the long bones, suggesting potential therapeutic benefits for additional aging-related bone loss disorders, such as osteoporosis.^13^

DEL-1 exhibits a key inhibitory effect on osteoclasts through its ability to repress the master regulator of osteoclastogenesis, namely the transcription of nuclear factor of activated T cells cytoplasmic 1 (NFATc1) by binding to Mac-1 integrin and elevating *Bcl6* expression.^30^ As a result, the modulation of DEL-1 expression by macrolides holds promise as a prospective approach to mitigate osteoclast activity for therapeutic purposes. Notably, clinical studies have reported favorable outcomes with the use of macrolides in the treatment of chronic periodontitis.^75^ This ERM-mediated DEL-1 expression has shown efficacy in inhibiting periodontitis progression and suppressing the expression of genes associated with osteoclast activity. Additionally, AZM effectively suppresses osteoclast resorptive activity and osteoclast formation by modulating NFATc1.^76^ This study reveals the capacity of macrolides and EM-523, in addition to ERM, to modulate DEL-1 expression and consequently influence osteoclasts. These macrolides, including EM-523, hold promise as potential agents with the dual capability of promoting bone regeneration while also suppressing osteoclastogenesis.

To exploit the immunomodulatory effects of macrolides, these antibiotics may need to be administered over an extended period of time. Therefore, there is a possibility that antimicrobial resistance may develop. This study indicates non-antibiotic ERM-derivative, EM-523, is an effective modulator of DEL-1 expression that promotes periodontal bone regeneration, which is a therapeutically important discovery. EM-523 effectively increased DEL-1 protein levels in the PDL and favorably modulated bone cells, osteoblasts, and osteoclasts, in lower effective concentrations than the macrolides tested. Therefore, EM-523 may have a more favorable safety profile and fewer adverse reactions (e.g., pseudomembranous colitis, as reported for erythromycin and other antibiotics),^77^^,^^78^ while additionally preventing antimicrobial resistance.

In summary, this study highlights the potential use of macrolide antibiotics and specifically EM-523 as potential therapeutic approaches for regenerating lost bone and preventing further disease progression, particularly in the aging population. Further understanding of the mechanisms and downstream effectors that underlie the immunomodulatory properties of macrolides and their derivatives can lead to the development of safe and effective clinical applications to improve bone regeneration in elderly patients with periodontitis and other inflammatory bone loss disorders.

### Limitations of the study

This study has revealed an increase in bone mass and changes in femoral bone parameters following ERM treatment by using the micro-computed tomography (micro-CT) and dual-energy X-ray absorptiometry (DXA). While our analysis involved these techniques, they were unable to offer the level of detailed information that could be provided by dynamic bone histomorphometry. Dynamic bone histomorphometry is a comprehensive methodology measuring bone remodeling at the level of individual events. It provides a quantitative means to assess alterations in bone microstructure, bone generation, and bone renovation, offering valuable insights into cellular events.^79^^,^^80^ By assessing parameters such as bone mineral density (BMD), trabecular thickness, osteoblast and osteoclast activity, and bone turnover rates, dynamic bone histomorphometry offers an in-depth understanding of cellular transformations occurring within bone tissue. Future studies should consider the incorporation of dynamic bone histomorphometry as it offers valuable insights into cellular transformations.

Our present work was performed in the mouse model and is thus uncertain whether the DEL-1-dependent beneficial effects of macrolides could be extrapolated to humans. Nevertheless, the ability of DEL-1 to inhibit periodontitis in mice was also confirmed in non-human primates.^81^ Moreover, DEL-1 expression is regulated similarly in humans and mice^82^ and DEL-1 performs similar functions (e.g., inhibition of neutrophil transmigration, osteogenesis and osteoclastogenesis) across species, probably because human DEl-1 has 96% and 99% sequence identity with its mouse and non-human primate counterparts, respectively.^28^^,^^71^^,^^81^^,^^82^^,^^83^ Macrolides are active in both mice and humans although it remains to be established whether macrolides, or the erythromycin derivative EM-523 can upregulate DEL-1 in human tissues as they do in mouse tissues.

## STAR★Methods

### Key resources table

**Table:** 

REAGENT or RESOURCE	SOURCE	IDENTIFIER
**Antibodies**		

Rabbit monoclonal anti-DEL-1 (clone EPR12451)	Abcam	Cat# ab190692
Rabbit polyclonal anti-alpha-smooth muscle Actin	Abcam	Cat# ab5694; RRID: AB_2223021
Goat polyclonal anti-CD31/PECAM-1	Novus Biologicals	Cat# AF3628; RRID: AB_2161028
Donkey anti-Rabbit IgG (H+L) Highly Cross-Adsorbed Secondary Antibody, Alexa Fluor™ 488	Thermo Fisher Scientific	Cat# A-21206 (also A21206); RRID: AB_2535792
Donkey anti-Goat IgG (H+L) Cross-Adsorbed Secondary Antibody, Alexa Fluor™ 594	Thermo Fisher Scientific	Cat# A-11058 (also A11058); RRID: AB_2534105
Mouse monoclonal to 6X His tag® (clone HIS-1)	Abcam	Cat# ab49936; RRID: AB_867459
Rabbit monoclonal anti-Histone H3 (clone D2B12)	Cell Signaling Technology	Cat# 4620; RRID: AB_1904005
Mouse monoclonal anti-C/EBPβ (clone H-7)	Santa Cruz Biotechnology	Cat# sc-7962; RRID: AB_626772
Rabbit IgG	Cell Signaling Technology	Cat# 2729; RRID: AB_1031062
Human IgG	Thermo Fisher Scientific	Cat# 02-7102; RRID: AB_2532958

**Chemicals, peptides, and recombinant proteins**		

Erythromycin (ERM)	TCI Chemicals	Cat# E0751; CAS# 114-07-8
Clarithromycin (CLR)	TCI Chemicals	Cat# C2220; CAS# 81103-11-9
Azithromycin (AZM)	TCI Chemicals	Cat# A2076; CAS# 117772-70-0
CLR ready-made solution in dimethyl sulfoxide	Sigma-Aldrich	Cat# SBR00011; CAS# 81103-11-9
EM-523	Kitasato University, Tokyo Japan^84^^,^^85^	CAS# 33396-29-1
Liberase solution	Merck	Cat# 05401119001
Cetylpyridinium chloride	Sigma-Aldrich	Cat# C0732; CAS# 6004-24-6
Alizarin Red S	FUJIFILM Wako	Cat# 100375; CAS# 130-22-3
ProLong™ Diamond Antifade Mountant with DAPI	Thermo Fisher Scientific	Cat# P36962
Recombinant mouse DEL-1 fused with human IgG1-Fc	This paper	Constructed following the previously described procedures with some modifications^30^
Phytone™ Peptone	Becton Dickinson	Cat# 211906
PEI MAX® - Transfection Grade Linear Polyethylenimine Hydrochloride	Polysciences	Cat# 24765; CAS# 49553-93-7
COSMOGEL(R) Ig-Accept Protein A	Nacalai tesque	Cat# 02179-14

**Critical commercial assays**		

Tartrate-resistant acid phosphatase staining kit	Cosmo Bio	Cat# PMC-AK04F-COS
Alkaline phosphatase (ALP) staining kit	Cosmo Bio	Cat# PMC-AK20
Masson trichrome staining kit	Abcam	Cat# ab150686
SimpleChIP® Plus Enzymatic Chromatin IP Kit (Magnetic Beads)	Cell Signaling Technology	Cat# 9005
SF Cell Line 4D-Nucleofector™ X Kit S	Lonza	Cat# V4XC-2032
Dual-Glo® Luciferase Assay System	Promega	Cat# E2920
SuperScript™ IV VILO™ Master Mix	Thermo Fisher Scientific	Cat# 11756050
Fast SYBR™ Green Master Mix	Thermo Fisher Scientific	Cat# 4385612
TaqMan™ Fast Advanced Master Mix for qPCR	Thermo Fisher Scientific	Cat# 4444557

**Experimental models: Cell lines**		

Male murine macrophage cells (RAW264.7 TIB-71)	ATCC	Cat# TIB-71; RRID: CVCL_0493
Murine osteoblastic progenitor cells (RCB1126: MC3T3-E1)	The RIKEN Bioresource Center	Cat# CVCL_0409
Male human periodontal ligament cells (hPDLCs)	Lonza	Cat# CC-7049
Human iPSC-derived mesenchymal stem cells (iCell® Mesenchymal Stem Cells)	Fujifilm Cellular Dynamics International	Cat #R1098
Female human embryonic kidney (HEK293) cells	ATCC	Cat# PTA-4488; RRID: CVCL_0045

**Experimental models: Organisms/strains**		

C57BL/6 mice	1. Charles River Laboratories (Kanagawa, Japan) 2. Jackson Laboratories (Bar Harbor, Maine, USA).	RRID:MGI:2159769
*Del1*^−/−^ C57BL/6 mice	Generated using gene editing by GEEP method by Setsuro Tech (Tokushima, Japan).^86^^,^^87^	N/A

**Oligonucleotides**		

guide RNA to generate *Del1*^−/−^ C57BL/6 mice	Choi et al.^88^	N/A
Primer: Mm_BamHI_Edil3: Forward 5′-ACTGCGGATCCATGAAGCACTTGGTAGCAGCC-3′	This paper	N/A
Primer: Mm_Edil3_Hs_IgG1-Fc: Forward 5′-CACTCTGCGGTCAGAGCTGCTGGGC TGCGCAGAGGAGGAACCCAAATCTTGTG ACAAAACTCACAC-3′	This paper	N/A
Primer: Hs_IgG1-Fc_Mm_Edil3: Reverse 5′-GGCACGGTGGGCATGTGTGAGTTTTGTC ACAAGATTTGGGTTCCTCCTCTGCGCAGC-3′	This paper	N/A
Primer: Hs_SphI_6xHis_IgG1-Fc: Reverse 5′-GTAGCGCATGCTTAGTGGTGATGGTGAT GATGTTTACCCGGAGACAGGGAGAG-3′	This paper	N/A
Primer: Hs_C/EBPβ binding sites of *EDIl3* promoter: Forward 5′-CTTATAGCAGAAGGAGCTGAAAGAG-3′	Maekawa et al.^31^	N/A
Primer: Hs_C/EBPβ binding sites of *EDIl3* promoter: Reverse 5′ TGGAGAACAATGAAGGCGTGAG-3′	Maekawa et al.^31^	N/A
Primers for qPCR to quantify gene expression in this study, see Table S1	This paper	N/A

**Recombinant DNA**		

pUC19 vector	Addgene	RRID:Addgene_50005
pEXA2J2 vector	Eurofins Genomics	N/A
pHEK293 Ultra Expression Vector I	Takara Bio	Cat# 3390
pGL3 firefly luciferase reporter plasmid	Promega	Cat# E1751
Human IgG1-Fc (IGHG-1; Accession: P01857, Pro100-Lys330)	Gang Deng et al.^89^	N/A
DEL1 promoter/luciferase reporter plasmid	Maekawa et al.^43^	N/A

**Software and algorithms**		

ZEN Blue imaging software	Zeiss	RRID:SCR_013672
ImageJ software	Schneider et al.^90^	RRID:SCR_003070
GraphPad Prism	GraphPad Software	RRID:SCR_002798

**Other**		

ZEISS Axio Imager 2 microscope	Zeiss	RRID:SCR_018876
EVOS M5000 microscope	Thermo Fisher Scientific	Cat# AMF5000
HS All-in-one Fluorescence Microscope	Keyence	Cat# BZ-9000E
Leica EZ4 Stereo Microscope	Leica	www.leica-microsystems.com
QuantStudio™ 3 Real-Time PCR System	Thermo Fisher Scientific	RRID:SCR_018712
GloMax®-Multi Detection System	Promega	Cat# E7041
Leica CM3050 S Cryostat	Leica	www.leicabiosystems.com
ScanXmate-A080	Comscan Techno	https://www.johnan.com/

### Resource availability

#### Lead contact

Further information and requests for resources and reagents should be directed to and will be fulfilled by the lead contact, Tomoki Maekawa (maekawa-t@dent.niigata-u.ac.jp).

#### Materials availability

• Plasmids generated in this study have been deposited to The Division of Microbiology and Infectious Diseases, Graduate School of Medical and Dental Sciences, Niigata University.
• Mouse lines generated in this study have been deposited to the animal facility of The Graduate School of Medical and Dental Sciences, Niigata University.
• Histological slides generated in this study belong to the collections of The Center for Advanced Oral Science, Graduate School of Medical and Dental Sciences, Niigata University and can be studied upon request.

#### Data and code availability

• All data reported in this paper will be shared by the lead contact upon request.
• This paper does not report original code.
• Any additional information required to reanalyze the data reported in this work paper is available from the lead contact upon request.

### Experimental model and study participant details

Animal experiments were approved by the Institutional Animal Care and Use Committee (IACUC) of Niigata University (approval no. SA60001) or by the IACUC of the University of Pennsylvania (approval no. 805528). Male C57BL/6 mice (RRID:MGI:2159769) were purchased from Charles River Laboratories (Japan, Inc., Kanagawa, Japan) or from Jackson Laboratories (Bar Harbor, Maine, USA). The mice were stored in ventilated cages individually and provided sterile food and water *ad libitum* under pathogen-free conditions. Mice were used for experiments at 18-month-old for aged mice. In some experiments (Figure 1), aged mice were 13-month-old. A DEL-1-deficient (*Del1*^−/−^) mouse strain was generated using gene editing by electroporation of Cas9 protein (GEEP) method by Setsuro Tech (Tokushima, Japan).^86^^,^^87^ The guide RNA was developed following a previous publication.^88^ Aged wild-type and *Del1*^*−/−*^ C57BL/6 mice (19 to 20-month-old) were intraperitoneally administered Erythromycin (ERM), Clarithromycin (CLR), or Azithromycin (AZM), or EM-523 (100 mg/kg for ERM and AZM; 25 and 20 mg/mL for CLR and EM-523, respectively). The control group was injected with 20% ethanol in PBS.

### Method details

#### Reagents

Macrolide antibiotics used in this study are as follows: ERM (TCI Chemicals, Japan), CLR (TCI Chemicals, Japan), and AZM (TCI Chemicals, Japan). The erythromycin derivative, EM-523, was provided by Kitasato University, Tokyo Japan.^84^^,^^85^ All reagents were used at previously reported doses^91^ and reconstituted as per the manufacturer’s protocols. Each substance was dissolved in 20% ethanol in PBS. For some *in vitro* experiments, CLR ready-made solution in dimethyl sulfoxide (Sigma-Aldrich, USA) was used.

#### Construction of recombinant mouse DEL-1 fused with human IgG1-Fc

Recombinant mouse DEL-1 fused with human IgG1-Fc (DEL-1-Fc) was provided by Dr. Toshihito Isono following the previously described procedures with some modifications.^30^ Total mRNA was isolated from RAW264.7 cells using TRI reagent (Molecular Research Center, Cincinnati, OH, USA) and cDNA was prepared from total RNA using a ReverTra Ace qPCR RT Master Mix with gDNA Remover (TOYOBO, Tokyo, Japan). The cDNA for mouse *Edil3*, the gene encoding DEL-1, including signal sequence was amplified and inserted into the BamHI-SphI site of the pUC19 vector (pUC19 *Edil3*). The positive clones were selected and confirmed by DNA sequencing by Eurofins Genomics (Tokyo, Japan). The cDNA encoding Pro100-Lys300 region of human IgG1-Fc (*IGHG1*; Accession: P01857) was synthesized and cloned into pEXA2J2 plasmid (pEXA2J2 *IGHG1*) by Eurofins Genomics. Using pUC19 *Edil3* and pEXAJ2 *IGHG1* as templates, *Edil3* and *IGHG1* were fused by overlap extension PCR to adda recognition sequence for BamH1 at the 5′ end of *Edil3* and polyhistidine tag, stop codon, and recognition sequence for SphI at the 3′ end of *IGHG1*. Then *Edil3* fused with *IGHG1* gene was inserted into the BamHI-SphI site of pHEK293 ultra expression vector I (Takara, Tokyo, Japan) and positive clones were selected and confirmed by DNA sequencing by Eurofins Genomics. HEK293T cells were transfected with this plasmid using PEI Max (Polysciences, Inc., Warrington, PA, USA). DEL-1-Fc was secreted into the culture medium (Dulbecco’s Modified Eagle’s Medium (FUJIFILM Wako Pure Chemical, Osaka, Japan) supplemented with 0.4 % phytone peptone (Becton Dickinson, Franklin Lakes, NJ, USA)) and was re-dissolved in PBS containing 300 mM L(+)-arginine after ammonium sulfate precipitation in order to avoid aggregation.^92^^,^^93^ DEL-1-Fc was purified using COSMOGEL Ig-Accept Protein A (Nacalai tesque, Kyoto, Japan) and the eluted DEL-1-Fc was dialyzed against PBS. The identity and purity of the protein were confirmed by Western blot analysis using anti-DEL-1 antibody (ab190692: Abcam, Cambridge, UK) and anti 6×His antibody (ab49936: Abcam). In the early experiments of this study (Figure 1), recombinant DEL-1-Fc generated and purified as previously described,^30^ comprised human DEL-1 fused with the human IgG1-Fc fragment. DEL-1 expression is regulated similarly in humans and mice and DEL-1 performs similar functions across species; human DEL-1 has 96% and 99% amino acid sequence identity with mouse and cynomolgus orthologs, respectively.^28^^,^^31^^,^^43^^,^^46^^,^^71^^,^^94^

#### Ligature-induced periodontitis (LIP)

We performed tooth ligation to induce periodontitis in the mouse model as we previously described.^41^ In brief, periodontal bone loss was induced by tooth ligation of the upper left 2^nd^ molar. The unligated (U) contralateral molar tooth was set as the baseline control. LIP was performed in 2-month-old and 12-month-old mice for ten days and the ligature remained intact or was removed after five days to enable resolution from periodontitis, as previously described.^27^ Mice were then sacrificed on day 10 or 15 for analyses.

#### Alveolar bone analysis

Alveolar bone changes were evaluated in maxillae by stereoscopic microscopy (Leica Microsystems, Wetzlar, Germany or Nikon Instruments, Melville, New York, USA). The distance from the cement-enamel junction to the alveolar bone crest (CEJ-ABC) was measured at six pre-established sites^95^ surrounding the ligated sites in the LIP model. Bone loss was calculated by subtracting the six-site CEJ-ABC distance for the ligated side of each mouse maxilla from the six-site total CEJ-ABC distance of the contralateral unligated side. Negative and positive values represent the mean bone loss and growth relative to the unligated, and untreated baselines, respectively. For naturally occurring periodontal bone loss in 19 to 20-month-old mice, the CEJ-ABC values were measured at 14 pre-established sites from the first molar to the third molar on both sides of the maxilla.^96^ Bone changes were calculated by subtracting the 14-site total CEJ-ABC distance for each mouse from the mean CEJ-ABC distance of the control group. In macrolide-treated mice, bone changes with positive values indicate bone growth relative to control-treated mice.

#### Analysis of bone phenotypes and femur measurements using microcomputed tomography (microCT) and DXA

Femoral bone samples were collected, and all the connective tissue was carefully removed. Bone mineral content (BMC) and bone mineral density (BMD) were measured by dual-energy X-ray absorptiometry (DXA) at Kureha Special Laboratory. MicroCT analysis was performed (ScanXmate-A080, Comscan Techno) to measure morphological indices in the distal metaphysis of femurs. Morphological indices were calculated in trabecular bones located 0.5 to 1.5 mm from the growth plates, using ImageJ software. Femurs were sectioned using the Kawamoto method^97^ and stained with Von Kossa, Toluidine blue, and Alcian blue.

#### Murine maxillary sections

The maxillae used for tissue sections were fixed in 4% paraformaldehyde phosphate buffer solution (Wako Pure Chemical Industries, Osaka, Japan) for 24 h, then decalcified in the Decalcifying Solution B (Wako Pure Chemical Industries) for one week at 4°C. Decalcified maxillae were then embedded in OCT Compound (Sakura Finetek, Torrance, California, USA), which was frozen in liquid nitrogen. Coronal sections (diameter = 12 μm/slide) were prepared using a cryostat (Leica Biosystems, Wetzlar, Germany).

#### Histological staining

An Alkaline Phosphatase (ALP) Staining Kit (PMC-AK20, Cosmo Bio) was used for detecting osteoblastic activity. ALP staining in each field was quantified and presented as a percentage of the ALP active region to the total area. The TRAP Staining Kit (PMC-AK04F-COS, Cosmo Bio) was used for detecting osteoclasts. TRAP+ multinucleated cells (≥ three nuclei) were photographed, and the number of positively stained cells per area were counted. A Masson’s trichrome staining kit (ab150686, Abcam) was used for detecting new bone formation. This staining technique stains mature bone and connective tissue blue, while staining immature new bone (osteoid) and collagen red.^27^ The histological images were obtained using a BZ-9000 Bio Revo all-in-one microscope.

#### Immunofluorescence staining (IF)

Murine maxillary sections were incubated with anti-DEL-1 primary antibody (ab190692, Abcam), anti-alpha-smooth muscle actin (ab5694, Abcam), anti-CD31/PECAM-1 (AF3628, Novus Biologicals). Secondary antibodies used in this study include Donkey anti-Rabbit IgG conjugated with Alexa Fluor™ 488 (A-21206, Thermo Fisher Scientific), Donkey anti-Goat IgG conjugated with Alexa Fluor™ 594 (A-11058, Thermo Fisher Scientific). All sections were counterstained with ProLong™ Diamond Antifade Mountant with DAPI (P36962, Thermo Fisher Scientific). All fluorescent images were obtained using a ZEISS Axio Imager 2 microscope and processed with ZEN Blue imaging software.

#### Primary mouse PDL cells

Primary mouse PDLCs were harvested and maintained as previously described.^98^ In brief, after euthanizing mice, the maxilla and mandible were carefully separated. The surrounding gingiva was removed prior to teeth extraction. A Leica (EZ4W) stereoscopic microscope (Leica Microsystems) was utilized during the extraction procedures. The collected teeth were washed with PBS and transferred to fresh wells. Liberase (Merck, 05401119001) solution was used at a final concentration of 2 Wünsch units/ml. The teeth were incubated in a digestion buffer inside 15 ml low-binding tubes (STEMFULL; Sumitomo Bakelite) and placed in an incubator at 37°C with shaking at 200 rpm. Subsequently, 10 ml of PBS with 2% fetal bovine serum (FBS) as a wash buffer was added, and the cells were centrifuged at 400 g for 8 mins at 4°C. The resulting pellet was resuspended in a washing buffer and passed through a 70-μm cell strainer. Following a second centrifugation at 300 g for 5 mins at 4°C, the cells were resuspended in a washing buffer and ready for downstream experiments.

#### Cell lines

The cell lines used in this study were murine macrophage cell lines RAW264.7 TIB-71 (ATCC), murine osteoblastic progenitor cell line MC3T3-E1 (The RIKEN Bioresource Center, RCB1126), human periodontal ligament cells (hPDLCs, CC-7049, Lonza) and human iPSC-derived mesenchymal stem cells (hMSCs, R1098, Cellular Dynamics International). All cells were cultured following the manufacturer’s protocols.

#### ChIP-qPCR assay

Enrichment of C/EBPβ binding at the *EDIL3* promotor region was evaluated in hPDLCs treated with or without macrolides and assessed using the SimpleChIP Plus Enzymatic Chromatin IP Kit with magnetic beads (Cell Signaling Technology) as described in previous studies.^43^^,^^99^ Briefly, cross-linked chromatin was immunoprecipitated with nonimmune rabbit IgG (Cell Signaling Technology) or anti–histone H3 rabbit IgG monoclonal antibody (mAb) (D2B12; Cell Signaling Technology) or anti-C/EBPβ mouse IgG (H-7; Santa Cruz Biotechnology, Dallas, Texas, USA). For RT-PCR, primers 5′ CTTATAGCAGAAG- GAGCTGAAAGAG 3′ and 5′ TGGAGAACAATGAAGGCGTGAG 3′ flanking 2 putative C/EBPβbinding sites in the *EDIL3* promoter (328 to 589 base pairs) were used. ChIP-qPCR data obtained using the anti-C/EBPβ antibody or nonimmune IgG were normalized using a percentage input method that normalizes chromatin input based on the following equation: % input = 100 × 2^Ct[adjusted input] – Ct[IP]^.^99^^,^^100^

#### Luciferase reporter assay

The luciferase reporter assay and construct of hDEL1 promoter/luciferase reporter plasmid (hDEL1-promoter-Luc) were described in our previous study.^43^ hPDLCs were cultured in 96-well plates at a density of 1 × 10^4^ cells per well and cotransfected with the hDEL1-promoter-Luc and pGL3 firefly luciferase reporter plasmid (Promega, Madison, Wisconsin, USA) as an internal transfection control using the 4D-Nucleofector V4XC for SF cell line (Lonza). hPDLCs were treated with ethanol-control, ERM, CLR, or AZM. Luciferase assay was performed using the Dual-Glo Luciferase Assay System and GloMax-Multi Detection System (Promega) according to the manufacturer’s instructions.

#### Osteoclastogenesis assay

Primary bone marrow-derived macrophages and RAW 264.7 cells were cultured to perform osteoclastogenesis assays as previously described.^41^ Histological images of TRAP^+^ MNCs were obtained using EVOS M5000 (Thermo Fisher Scientific). The number of TRAP^+^ MNCs were counted. The TRAP^+^ area was photographed and measured (as a percentage of TRAP^+^ area relative to the total area) using ImageJ software (V.1.53).

#### Osteoblastic progenitors

Primary cultures of osteoblastic progenitor cells were derived from 3-day-old *Del1*^*−/−*^ mice by digesting calvarias in PBS containing 0.1% collagenase Type I (Worthington) and 0.2% ROCHE Dispase II (MilliporeSigma) for 20 min at 37°C. The digestion was sequentially performed three times, and cells isolated from the digestions steps were cultured in a-MEM medium supplemented with 10% FBS.

#### Osteoblast differentiation assay

hMSCs, MC3T3-E1 cells, and primary osteoblastic progenitor cells were used in a Modeling Osteoblast Differentiation assay to study the effects of administered substances on the osteogenic differentiation potential of mesenchymal stem cells as described in the manufacturer’s protocol. In brief, hMSCs, MC3T3-E1 cells and primary osteoclastic progenitor cells were cultured in osteoblast differentiation medium after seeding and cell attachment, then treated with the control solvent or macrolideswhen the cells reached 80% confluency. After two weeks of culture in osteoblast differentiation medium, the media were replaced with osteoblast mineralization medium, with or without the control solvent or macrolides, for one week. All media were replaced every two days.

#### Semi-quantification of calcification nodules from osteoblasts

Mineralized bone nodules from differentiated osteoblasts were visualized by staining with 2% Alizarin Red S (FUJIFILM Wako, Japan) in distilled water with pH 6.3-6.4. To visualize the calcium nodules, the cultures were washed twice with PBS, fixed with 10% neutral buffered formalin for 15 min, and washed again with distilled water. Alizarin Red staining was performed by adding 2% Alizarin Red S solution at RT for 45 min. After washing with distilled water to remove the excess dye, the wells were photographed using a stereoscopic microscope (Leica Microsystems, Wetzlar, Germany) and measured (as a percentage of calcified area relative to the total area) using the ImageJ software (V.1.53). To quantify the calcified nodules from osteoblasts, each sample was de-stained in 10 mM sodium phosphate containing 10% cetylpyridinium chloride (Sigma-Aldrich), pH 7.0 for 15 min at 37°C. The absorbance was measured in triplicates at 560 nm using a plate reader.

#### Quantitative real-time PCR (qPCR)

Total RNA was extracted from mouse maxillary palatal gingiva and cultured cells using TRI reagent (Molecular Research Center, USA). RNA was quantified using Nanodrop One (Thermo Fisher Scientific, USA). RNA samples were reverse transcribed using SuperScript VILO IV Master Mix (Thermo Fisher Scientific, USA), and qPCR was performed using the QuantStudio™ 3 Real-Time PCR System (Thermo Fisher Scientific, USA). The RNA was quantified using the comparative CT method, which uses a reference sample and an endogenous control to determine the relative expression of the target gene.

### Quantification and statistical analysis

After confirming normal distribution of the data, we analyzed the mean between groups using one-way ANOVA and post-hoc tests. All statistical analyses were performed using GraphPad Prism Version 9.4.1 (458). (GraphPad Software, Inc., La Jolla, CA, USA). p-values of < 0.05 were considered statistically significant. Asterisks shown in the results section correspond to ∗*P* < 0.05, ∗∗*P* < 0.01.
