# Supplementary material for: A novel macrolide–Del-1 axis to regenerate bone in old age
Source: iScience. 2024 Jan 4;27(2):108798. doi: 10.1016/j.isci.2024.108798 (PMC10797555; doi:10.1016/j.isci.2024.108798)
Supplement: Document S1. Figures S1 and S2 and Table S1 [file mmc1.pdf]

iScience, Volume *27*

Supplemental information

A novel macrolide–Del-1 axis

to regenerate bone in old age

Kridtapat Sirisereephap, Hikaru Tamura, Jong-Hyung Lim, Meircurius Dwi Condro Surboyo, Toshihito Isono, Takumi Hiyoshi, Andrea L. Rosenkranz, Yurie Sato-Yamada, Hisanori Domon, Akari Ikeda, Tomoyasu Hirose, Toshiaki Sunazuka, Nagako Yoshiba, Hiroyuki Okada, Yutaka Terao, Takeyasu Maeda, Koichi Tabeta, Triantafyllos Chavakis, George Hajishengallis, and Tomoki Maekawa

REAGENT or RESOURCE	SOURCE	IDENTIFIER
Oligonucleotides		
Gapdh (Mm99999915_g1)	Thermo Fisher Scientific	Cat# 4331182
Del1(Edil3) (Mm01291247_m1)	Thermo Fisher Scientific	Cat# 4331182
Il10 (Mm01288386_m1)	Thermo Fisher Scientific	Cat# 4331182
Il17a (Mm00439618_m1)	Thermo Fisher Scientific	Cat# 4331182
Il1b (Mm00434228_m1)	Thermo Fisher Scientific	Cat# 4331182
GAPDH (Hs02786624_g1)	Thermo Fisher Scientific	Cat# 4331182
DEL1(EDIL3) (Hs00964112_m1)	Thermo Fisher Scientific	Cat# 4331182
RUNX2 (Hs01047973_m1)	Thermo Fisher Scientific	Cat# 4331182
SP7 (Hs01866874_s1)	Thermo Fisher Scientific	Cat# 4331182
BGLAP (Hs01587814_g1)	Thermo Fisher Scientific	Cat# 4331182
GAPDH (NM_002046)	OriGene Technologies	Cat# RC202309
ACTA2 (NM_001613)	OriGene Technologies	Cat# HP205437
Primer: Mm_16s RNA: Forward<br>5'- ACTCCTACGGGAGGCAGCAGT-3'	Integrated DNA Technologies	Ref# 108621999
Primer: Mm_16s RNA: Reverse<br>5'- ATTACCGCGGCTGCTGGC-3'	Integrated DNA Technologies	Ref# 108622000

Table S1. Primers for qPCR to quantify gene expression in this study, related to the STAR Methods.

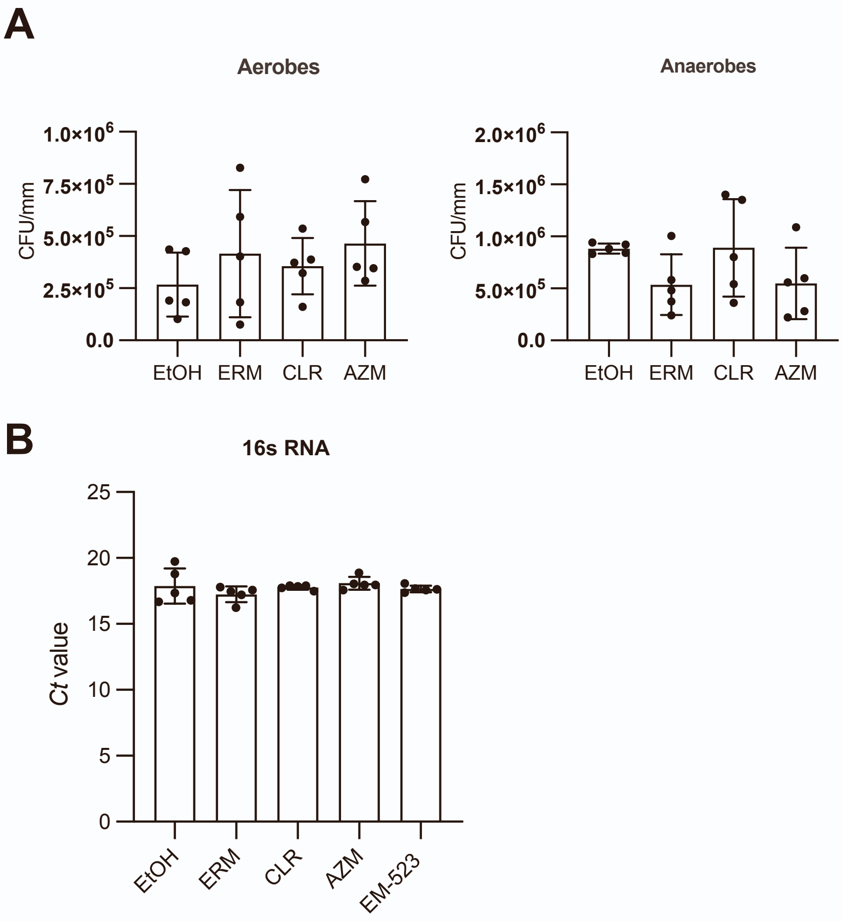

Figure S1. Intraperitoneal injection of macrolides does not affect the microbes attached to the ligatures, related to Figure 2.

(A) After nine days of ligature, the silk ligatures with 4 mm lengths from each ligated group were removed and vortexed with PBS. The serial dilution of the microbial-mixed solution was performed and plated on blood sheep agar (BSA) for 24 h with aerobes and anaerobic conditions. The colony-forming unit (CFU) per 1 mm ligature was calculated for each sample to represent the number of aerobic bacteria attached to the silk ligature. **(B)** The Ct values of 16s RNA gene expression from ligatures were quantified by qPCR on day nine of the ligature period. Data are mean ± S.D. (error bars) (*n* = 5 silk ligatures per group) (one-way ANOVA and Dunnett's post-test). NS (Not significant, not shown in this graph) compared with the EtOH-control group.

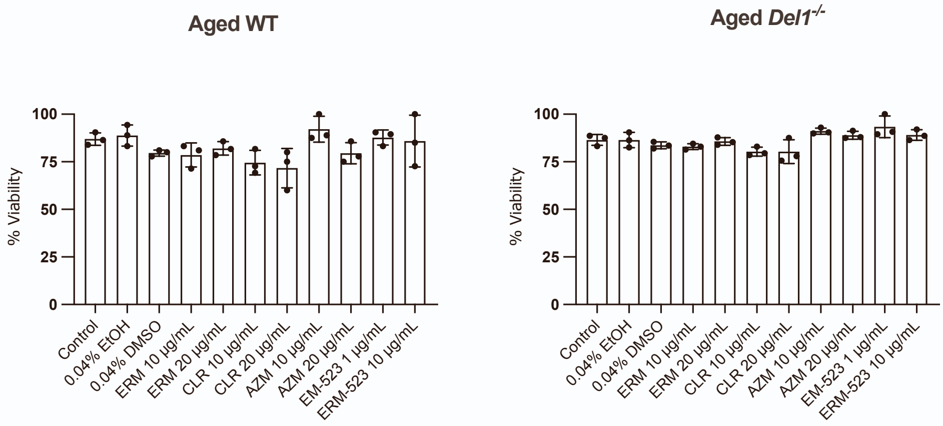

Figure S2. Macrolide and EM-523 treatment does not affect cell viability of primary mouse bone marrow-derived macrophage cells, related to Figure 4.

Cell viability of primary mouse bone marrow-derived macrophages collected from **(left panel)** aged WT and **(right panel)** aged *Del1*$^{-/-}$ mice after treatment with control solvent or indicated macrolide treatment. Trypan blue exclusion test was performed to access cell viability after treatment with control solvents or indicated macrolide for 72 h. Data are mean ± S.D. (n = 3 sets of cultures/group) one-way ANOVA and Dunnett's post-test. NS (Not significant, not shown in this graph) compared with the EtOH-control group.
